# Hearing the Unheard: An Interdisciplinary, Mixed Methodology Study of Women’s Experiences of Hearing Voices (Auditory Verbal Hallucinations)

**DOI:** 10.3389/fpsyt.2015.00181

**Published:** 2015-12-23

**Authors:** Simon McCarthy-Jones, Maria Castro Romero, Roseline McCarthy-Jones, Jacqui Dillon, Christine Cooper-Rompato, Kathryn Kieran, Milissa Kaufman, Lisa Blackman

**Affiliations:** ^1^Department of Psychiatry, Trinity College Dublin, Dublin, Ireland; ^2^Department of Cognitive Science, ARC Centre of Excellence in Cognition and Its Disorders, Macquarie University, Sydney, NSW, Australia; ^3^School of Psychology, University of East London, London, UK; ^4^Independent Scholar, Dublin, Ireland; ^5^Department of English, Utah State University, Logan, UT, USA; ^6^Hill Centre for Women, McLean Hospital, Boston, MA, USA; ^7^Department of Psychiatry, Harvard Medical School, Boston, MA, USA; ^8^Department of Media and Communications, Goldsmith’s College, London, UK

**Keywords:** abuse, gender, hallucinations, psychosis, schizophrenia, traumatology, women, women’s health

## Abstract

This paper explores the experiences of women who “hear voices” (auditory verbal hallucinations). We begin by examining historical understandings of women hearing voices, showing these have been driven by androcentric theories of how women’s bodies functioned leading to women being viewed as requiring their voices be interpreted by men. We show the twentieth century was associated with recognition that the mental violation of women’s minds (represented by some voice-hearing) was often a consequence of the physical violation of women’s bodies. We next report the results of a qualitative study into voice-hearing women’s experiences (*n* = 8). This found similarities between women’s relationships with their voices and their relationships with others and the wider social context. Finally, we present results from a quantitative study comparing voice-hearing in women (*n* = 65) and men (*n* = 132) in a psychiatric setting. Women were more likely than men to have certain forms of voice-hearing (voices conversing) and to have antecedent events of trauma, physical illness, and relationship problems. Voices identified as female may have more positive affect than male voices. We conclude that women voice-hearers have and continue to face specific challenges necessitating research and activism, and hope this paper will act as a stimulus to such work.

## Introduction

Hearing a voice with a compelling sense of reality, yet in the absence of a corresponding external stimulus, is part of our heritage (1). While termed auditory verbal hallucinations by contemporary science, referring to such experiences as “hearing voices” has been argued to be less colonizing of people’s experience (2). In the mid-twentieth century, voice-hearing was most commonly associated with schizophrenia, being a central part of its diagnostic criteria and as a result found in around 60–70% of people given this diagnosis (3). The turn to a symptom- or complaint-based approach to schizophrenia (4) led to a research focus on voice-hearing specifically. Hearing voices is now recognized as occurring trans-diagnostically, including in people diagnosed with post-traumatic stress disorder, dissociative disorders, and borderline personality disorder (5). It is also found outside of psychiatric contexts, with a low single digit percentage of the general population having extended voice-hearing experiences without meeting criteria for psychiatric diagnoses (6, 7), evidencing that the experience is not necessarily associated with dysfunction or impairment. Toward the end of the twentieth century, the development of an influential movement of voice-hearers and their allies, the Hearing Voices Movement (HVM), solidified voice-hearing not only as an important research topic, but as a social and civil rights issue (8).

This paper explores voice-hearing from a gender perspective, which has always been, regrettably, marginal to mental health research (9). We approach this in three ways – historically, qualitatively, and quantitatively – all of which bear on the roles of sexism, sexist exploitation, and oppression [cf., Ref. (10)] in the causes, content, and consequences of women’s voice-hearing. We then attempt triangulation and synthesis of this experimental tripartite, interdisciplinary approach, in an entanglement that attempts to create perspectives and conclusions that the performance of these studies in isolation would not permit.

## A Brief History of Women Hearing Voices

Historically, understandings of women’s experiences of voice-hearing have been driven by patriarchal theories of how women’s bodies function. Women’s physical makeup was believed to make them more prone to “contaminations” and “impressions” (11), and to render them unsuitable for both reasoning and giving reliable testimony, requiring their voice-hearing experiences to be interpreted by men. Men used the extent to which women’s behavior, appearance and demeanor adhered to prescribed gender roles as a way to invalidate their voice-hearing, when expedient. While aspects of this are still seen today, a major shift in understanding has been to conceptualize the mental violation of the mind represented by some women’s experiences of voice-hearing as, in many cases, a direct consequence of the physical violation of their bodies.

### Early Voice-Hearing Experiences of Women

The earliest detailed records of women’s voice-hearing resulted from the medieval interest in preserving religious experience. Medieval medical understandings about men’s and women’s bodies shaped the way the two sexes were expected to perceive the divine and, thus, the ways they heard voices. From the Greeks to the European Enlightenment, humoral theory held sway. This was the belief that the healthy body had four humors in correct proportion. In this, men’s bodies were hot and dry, ideal conditions for intellectual development, whereas women’s were moist and cold, making them less suited to intellectual development, and more governed by their bodies’ need to seek out equilibrium through sexual contact with men’s warm dryness (12). As such, men were associated more closely with the intellectual and women more closely with the body. In a religious setting this meant that men could more easily approach God through *scientia* or intellectually derived knowledge gained through traditional education (e.g., scriptural study), whereas women could approach the divine through *sapientia*; felt/intuited knowledge infused by God. The association of women with the senses rather than the intellect made them the perfect conduits for heavenly voices and experiences, but also rendered them more vulnerable to accusations of invasions of the demonic [see Ref. (13)].

Scholars of medieval and gender history [e.g., Ref. (14, 15)] have argued that medieval women visionaries took advantage of such assumptions about women’s physical nature. These visionaries took what could be seen as a limitation (a perceived lack of ability to gain *scientia*) and turned it to an assertion that they were more bodily connected to God than men and, therefore, could experience the divine more easily through their senses, establishing their authority to speak of their own visions and auditions. This authority could still be questioned by male clerics. Jean Gerson (d. 1429) insisted that “every teaching of women, especially that expressed in solemn word or writing, is to be held suspect, unless it has been diligently examined” [Ref. (16), p. 40]. All medieval people claiming to hear God’s voice were subject to discernment, or steps to determine the nature and origin of the voice; women, however, had an extra hurdle to cross because of suspicions about their biological, intellectual, and moral nature (16).

Although medieval women were allowed to be recipients of divine voices, their experiences were defined and interpreted by the male-dominated Church. Women’s accounts of voice-hearing were directly or indirectly shaped by male intermediaries as they had to be written down by literate people. This generally limited scribing to men, because women were mostly barred from universities and only a minority of educated women outside the convents knew Latin. There is debate over how much editorial control male clerics had over the shaping of women’s accounts. Hildegard of Bingen (d. 1179) heard divine voices and recorded them (or had them recorded) while firmly asserting her illiteracy, allowing her to assert she was merely the *channel* of the divine message (17). Voice-hearing women and their scribes make this point repeatedly: that the women are illiterate and their prophetic speech needs to be recorded by a learned male [e.g., Ref. (18)]. While the growth of vernacular voice-hearing throughout this period was influential and liberating, communications in Latin remained important because they offered women access to a realm from which they might have otherwise been (at least partially) excluded.

Historical records [e.g., Ref. (19–21)] are limited to women’s voice-hearing considered to be of divine origin and, therefore, worth committing to parchment (i.e., saints), leaving us few accounts of ordinary women hearing voices. It is only in the fifteenth century, in the autobiography of Margery Kempe (d. 1438), that we first get access to the writings of a woman whose claims of her own experience were rejected by the Church (22). In the accounts we do have, women are often justifying why, in a male dominated society, God should be speaking to women. Hildegaard was forced to justify her prophetic mission as a woman by arguing that God could no longer rely on corrupt men and had turned to communicating with frail women such as herself (23). The acceptance of her voices and visions as authentic was in part due to her positioning herself as a humble woman treading on forbidden ground.

This theme is also seen in the early modern period. Teresa of Avila (d. 1582) had to acknowledge and tactically supplicate herself to the Church’s patriarchy. Under the guise of this assumed humility, she was able to successfully implement radical reforms of the Carmelite nuns, such as insisting that nuns should choose their own Spiritual Directors. In contrast, Lady Eleanor Davies (d. 1652) took a more forthright approach, and in explaining why a member of the “weaker sex” was hearing God’s voices, suggested this was due to the stubbornness and stupidity of men in her generation (24). This, when added to the political nature of her revelation, led to her being committed to Bethlam (24).

Voice-hearing could serve as a way for medieval women to make societal criticisms that their own voices alone would not be given the authority to convey. For example, Margery Kempe used God’s voice as a screen for social criticism (25) and was able to do this by drawing on both the recognized format of the holy woman who renounces early social and sexual roles (25) and the genre of female sacred biography (26). Similarly, de Beauvoir (27) argued that medieval women such as Joan of Arc (d. 1431), and early modern women such as Teresa of Avila, had visions and voices which “simply provide objective images for their certitudes” (p. 678), encouraging them to persist with the goals they have set. Voices could hence give women authority to pronounce on matters beyond those allotted to them by men.

The authentication of the source of a voice as divine – in addition to being adjudged by factors such as whether it was consistent with scripture and dogma – was in part assessed by a moral evaluation of the recipient and the extent to which their behavior was consistent with gendered norms (16). This bore particularly heavily on women, who were not part of the evaluation process. Men assessing the voice took into account the perceived blessedness or piety of the recipient and her behavior. In the case of women, heightened importance was given to those virtues deemed gender appropriate, like patience, humility, and obedience to a male spiritual advisor (16). This was especially important for women living “in the world” as laywomen.

Medieval discernment was greatly shaped by a woman’s sex, but did not end there. Class, social standing, religious occupation and perceived character also factored heavily into how easily her voices were accepted as authentic manifestations of God. For example, Margery Kempe was criticized for being a prideful lay woman, for abandoning her husband, and for dressing in white as if she were a virgin (22). Women whose voices were most suspect appear to have been the lay, peasant-class or merchant-class women who were not closely affiliated with religious orders [e.g., Christina Mirabilis, see Ref. (20)].

Despite the potential for women who heard voices in the late medieval period to be both validated and valorized, by the early sixteenth century the Church’s theological and social anxieties led such women to be viewed with increasing antipathy, as heretics, witches, victims of possession or fakes (11). As the early modern period ended, and psychiatry began, focus shifted to a secular medical understanding of women’s voice-hearing.

### Women and Voice-Hearing After the Birth of Psychiatry

After centuries of struggle between medicine and religion, hegemonic control of voice-hearing passed to medicine, via psychiatry, in the nineteenth century. The term “psychiatry” (psyche, soul/life; -iatry, physician) was first used by Reil in 1808, who emphasized that people who were mentally ill should be not be treated by experts of other disciplines (such as psychology or theology), but by a new type of doctor, the psychiatrist (28). Medicine wanted women; Ferry [1870, as cited in Ref. (29)] argued that “women must belong to science or else they will belong to the church” (p. 374), and that medicalizing women’s distress was desirable as this meant they consulted a physician rather than a priest (29).

In a fashion similar to the experiences of medieval women’s reliance on male scribes, the practice of medicine relied upon male physicians writing case histories that explicated the causes and cures for women’s ailments like hysteria. Unfortunately, despite encountering clear evidence of child and adult sexual abuse in a large number of female patients, many early psychiatrists refused to recognize this as causal of experiences such as voice-hearing (30). Although the famed neurologist Jean-Marie Charcot came to allow a role of trauma in causing “hysteria,” his focus was on the body and on finding a lesion associated with the condition. He could pass over such patients’ earlier experiences of being raped “cursorily and inaccurately” (31), and famously paid little attention to hysteria patient’s words, treating them as vocalization not communication (32). There was hence little chance of Charcot making potential links between earlier traumas and the content of voice-hearing even when some of his famous patients, such as Rosalie Leroux, had experience of both (31). In addition, Charcot used the bodies of women diagnosed with hysteria, via public demonstration and photography, for teaching, diagnostic, and promotional purposes (31, 33). Today, it is difficult to view this as anything other than exploitation with a degree of sexualization.

Many of Charcot’s students tried to find the cause of hysteria, which necessitated talking to female patients. The result was, as Herman (34) puts it, that “for a brief decade men of science listened to women with a devotion and respect unparalleled before or since” (p. 12). As a result, Freud initially claimed a relation between child sexual abuse and voice-hearing, but then retracted it (35). A range of theories exist for why he did this; personal fears, professional fears, and unwillingness to face down patriarchal hegemony are all theorized (30, 33). In contrast, another student of Charcot’s, Janet, made significant strides toward honoring the lived experiences of women through his influential writings on dissociation, explicitly relating trauma to voice-hearing through this mechanism (36, 37).

Although the First World War led to wider recognition that some mental disorders could have their roots in traumatic events, this focused on individuals with combat trauma. It was not until much later in the century that, as Herman (34) puts it, “it was recognised that the most common post-traumatic disorders are those not of men in war but of women in civilian life” (p. 28). Trauma’s centrality came to prominence again in the 1960s in the work of people such as R. D. Laing, who highlighted how voice-hearing could be an intelligible experience, and not simply the outpouring of a diseased brain (38). This highlighted that to only apply a model of endogenous mental disorder to voice-hearing would be to conceal and tacitly exonerate the abuse many hearers had suffered.

It was only in the 1970s, due to the women’s movement, that a greater awareness was created of the extent of trauma against women, particularly sexual assault (34). Feminists defined rape as a crime of violence, rather than a sexual act, and as a method of political control, subordinating women through terror (34), with Brownmiller (39) arguing it was a “conscious process of intimidation by which all men keep all women in a state of fear” (p. 15). In 2011, the US National Intimate Partner and Sexual Violence Survey (40) found that 18.3% of women reported being raped in their lifetime (compared to 1.4% of men). Of these women, 42% experienced their first rape before the age of 18. Thirty-five percent of women raped before the age of 18, were also raped as an adult. Ninty-eight percent of female rape victims reported only male perpetrators (with 93% of male rape victims reporting only male perpetrators). This growing awareness of the prevalence of childhood sexual abuse and adult rape, largely by men, was a crucial step in illuminating the link between such violations and women’s experiences of madness and distress, as well as making visible the structural barriers to acknowledging and responding appropriately to such transgressions.

Today medicine is able to provide interventions, such as dopamine blockers [“antipsychotic medication”; (41)], and explanations, such as a biomedical model, that prove satisfactory and helpful to some women who hear voices. Yet for others, medicine has continued to define their experiences in a colonizing fashion (2), and still functions to obscure the role of adverse life experiences and the emotions they may cause, which are now recognized as a common cause of voice-hearing (see below). Many studies of women and madness already demonstrate how patriarchy has reinterpreted women’s anger, suffering, and oppression into signs of mental pathology [e.g., Ref. (42)] and feminists have shown how diagnoses may be suffused with the dominant ideologies of their time and place and used to regulate behavior (43).

Documentation of the relation between trauma/abuse and voice-hearing is now extensive [e.g., Ref. (44–46)]. Bentall et al. (44) found that being raped increased the odds of voice-hearing sixfold. Janssen et al. (47) found child abuse prospectively predicted the development of voice-hearing, even after controlling for a family history of psychosis. This opens up an understanding of women’s voice-hearing not as a symptom of madness or disease, but of a patriarchal society that attempts to control women’s bodies. It can be seen as an inevitable result of what Hooks (10) describes as patriarchal thinking: the embrace of an ethics of domination, which says the powerful have the right to rule and can use any means to subordinate others, with both men and women abusing children, either physically or emotionally. In a rape culture (48), voice-hearing is inevitable.

While some women may be able to situate and contextualize their voices and potential experiences of trauma (49), other women who hear voices, due to the kinds of repressive practices of marginalization touched on above, have either been prevented from listening to their voices and articulating their concerns, or are simply unable due to engrained, embodied and entrenched experiences of oppression and institutionalized practices of silencing, shaming, and forgetting. Such consequences can be seen in Grace Cho’s (50) auto-ethnography of her experience of growing up with a mother who heard voices. Cho’s mother migrated to the USA at the end of World War Two as a GI bride, but never spoke about her migration story. What surrounded it were patterns of shame, secrecy, and silence. Cho attempted to reconstruct what her Mother’s voices might be communicating about other possible pasts that cannot or have not been told, and did not dismiss her Mother’s voices as irrational perceptions but rather approached them as ghostly interlocutors from the past, which are meaningful. She documented how an unspoken history can become transmuted into a hallucination and haunt the next generation, being carried in fragments, traces and murmurings by voices, waiting to be deciphered by those willing and able to “listen” to what they might have to say (51, 52).

### The Emergence of the Hearing Voices Movement

In the same way that feminists made explicit the complex, multi-factorial ways in which women’s personal experiences of oppression and discrimination operate within a wider social and political context, activists within the HVM have applied the concept of “The Personal is the Political” to their experiences. In the same ways in which women’s experiences of oppression and discrimination were limited, molded and defined by the broader social and political context, the same is argued to be true of experiences of madness and distress (53). The HVM is a prominent mental health service-user/survivor movement that promotes the needs and perspectives of experts by experience (i.e., voice-hearers themselves) in the phenomenon of hearing voices (54), in conjunction with allied mental health professionals (experts by training). Parallels exist between the HVM and the feminist movement: both operate within a broad framework of collective protest, solidarity, and a demand for social and political reform. As Hornstein (55) has remarked, “although not explicitly framed as ‘feminist’, this approach has had special appeal to the countless thousands of women who have finally had the reality of their own experiences – especially of sexual violence – taken seriously and used as a source of insight into what might actually help them” (p. 34).

Just as services for survivors of abuse and violence were originally organized outside of the traditional mental health system, often with the assistance of professional women inspired by such alternatives (34), much of the work of the HVM has taken the same route (8). Just as feminists first met together to share commonalities of experience – in the form of consciousness raising groups, support groups for survivors of abuse and sexual violence and so on – and then to mobilize to take a stand against their collective oppression, voice-hearers began to organize themselves and to form a collective identity. A crucial component of the formation of this collective identity has been the documentation, dissemination and public sharing of testimonies from activists within the HVM [e.g., Ref. (49)]. As feminist activist, Kali Tal (56) states: “When a survivor testifies, she both purges herself of an internal ‘evil’, and bears witness to a social or political injustice” (p. 200).

For the people who initiated the first Hearing Voices Group, simply talking to each other about stigmatized experiences, which had previously been silenced, censored, or led to the loss of their liberty or forced medication (a common result of disclosure in traditional psychiatric settings), was a revolutionary act. Through the sharing of experiences previously relegated to aberrant symptoms in need of eradication, the vital role of agency, self-determination, and collective action emerged. The realization that voices are frequently a consequence and articulation of taboo and trauma that wider society does not acknowledge, and that diagnosis adds insult to injury in concealing such injustices – with the additional ignominy of an imposed, stigmatized identity – led to marginalized individuals becoming self-determined and empowered allies and, ultimately, to the formation of a worldwide HVM (8).

For many voice hearers, social action is a key part of recovery. Third wave feminism introduced the concept of intersectionality to describe the ways in which oppressive institutions (e.g., sexism, racism, classism, homophobia, and transphobia) are interconnected and cannot be examined separately from each other (57). Similarly, the HVM sees it as imperative to refute the individualized and pathologising framing of understandable reactions to devastating experiences and, instead, to actively and collectively question the orthodoxy, challenge the *status quo* and address multiple, oppressive political structures. This vital process enables a transformation of a stigmatized experience from a position of isolation, shame and victimization to one of collective empowerment (58). As a result of the HVM’s research and campaigning, mental health services for women who hear distressing voices are now increasingly considering trauma-informed approaches, and are opening to HVM-inspired approaches when women do not find traditional medical frameworks are a good fit with their experiences.

In order to better understand the experiences of women hearing voices today, and to explore how they define their experiences, we next report results of a qualitative investigation into this question.

## A Qualitative Study of Women Hearing Voices

### Method

#### Participants

Inclusion criteria were that participants were female, aged 18–65 with personal experience of voice-hearing. Exclusion criteria were being non-English speakers or deemed too acutely unwell by keyworkers. Participants were recruited through mental health services, the English Hearing Voices Network, and a local mental health charity. In the invitation letter for the study, participants were informed that the study was of “women’s experiences of hearing voices and of the significant relationships in their lives… [and] your experience more generally in society, for example, in terms of social life or employment.” Participants from mental health services were gendered by keyworkers who identified women who they thought could be suitable, and those recruited from other sources were self-identified as women (being a transwoman was not a listed exclusion criterion for the study).

Eight women participated. Four were aged in their late twenties or early thirties, three were aged around forty, and one was in her fifties. Three were White-English, one White-Scottish, one White-Irish, one White-Australian, one African-Caribbean, and one African. Half defined themselves as working class and half as middle class. Four were in long-term relationships. None had children, one was currently in full-time waged employment, and all but one currently heard voices. All had previously been admitted to psychiatric units with five currently taking some form of medication for their voices and still being in contact with a mental health professional.

#### Methodology and Procedure

Interpretative phenomenological analysis [IPA; (59)] was selected as the most appropriate methodology. IPA explores individuals’ understandings of experiences through systematic analysis of first-hand accounts, assuming a hermeneutic-ontological phenomenology, which moves beyond the subject-object divide, understanding “reality” as repeatedly co-constructed but also given to us and, thus, people as making sense of their experiences “in relation” (60, 61). With participants’ informed consent, face-to-face interviews were undertaken using a semi-structured interview. The interview covered four different aspects of participant’s lives: general background, significant relationships, experiences of hearing voices, and the experience of being a voice-hearing woman in contemporary Britain. Interviews (by Maria Castro Romero) took around 90 min each to complete, and were audiotaped and transcribed for the purposes of analysis; all identifying details were changed to protect participants’ identities. The researcher also took notes on non-verbal aspects of the interview and her own thoughts and feelings immediately after each interview. Ethical approval to carry out this study was granted from the North Essex Mental Health Partnership NHS Trust Research & Development Department and Ethics Committee, and the Ethics Committee of the University of East London.

#### Data Analysis

Following Smith et al. (59, 62), participants’ narratives were analyzed through a circular process of interpretation involving three stages: the phenomenological phase; the interpretative phase; and the integrative phase. Finally, themes from each interview were consolidated to produce commonalities and controversies. A narrative was constructed around the super-ordinate and sub-ordinate themes, ensuring that each could be clearly linked to specific extracts from the transcripts.

#### Trustworthiness

A number of procedures were followed to attempt to ensure the reliability and validity of the analyses, based upon Elliott et al.’s (63) criteria for assessing the quality of qualitative research. The researcher’s (Maria Castro Romero) own values and beliefs are disclosed in order to allow the reader to understand potential biases in the analyses: assumptions about women’s generally subordinate position in our society; the influence of structural and contextual factors in connection to gender and distress; sensitivity to issues of power and inequality; and a desire to provide a non-pathologising context (e.g., respecting the women’s own frames of reference, rather than being led by a theoretical position).

### Results

Three main super-ordinate themes and sub-ordinate themes of relevance to the current paper are summarized here (we note that this artificial organization is used for clarity and ease of reading, but that it cannot fully do justice to the complexity of the accounts provided by participants).

#### Theme 1: The Experience of Hearing Voices

##### Sub-Theme I: The Voices

All women described hearing mainly hostile, critical, and angry voices. They were experienced as shocking and distressing partly because they often used abusive language, distinct from their own daily vocabulary (Sue: “I heard someone shouting at me, something along the lines of ‘what the fuck are you doing at this hour of the day, shouldn’t you be at work’”). There was also some evidence that distress was exacerbated by the perception that the voices were not simply issuing generic abuse, but were working diligently and intelligently to upset the hearer and using differing strategies to achieve this aim. These included interpersonal violence, disrespect, relentlessness, veiled threats, and more indirect and sophisticated psychological undermining or devaluing. For example, Helen described how a male voice took a patronizing stance, “he” had “sworn once or twice, but as a rule he’s far more sort of, he’s almost too polite [laughter], you know, when someone is being ultrapolite to try and get to you more, very clipped and talking very slowly as though they’re talking to a child and things like that.”

Five of the women heard benevolent voices, which seemed to serve the function of protecting them against the negative comments of other voices, using a reassuring, caring manner (Kim: “always had this voice in my head that tells me I’ll be alright, and it feels almost like a sort of guardian angel looking after me”). The positive voice was often described by the women as being their own voice. However, benevolent voices were not always welcomed, as they were seen as part of an unwanted experience, as part of being ill and out of the women’s control.

When asked who their voices were like, some were able to make direct matches (Kim: “The bad voice is my brother – purely my brother”). Others made matches on content, which could overrule the gender of the voice (Sue: “I don’t think the male voices have to do with [father]. I think all of them…in some respects, are like my mother”). Others said the voices were simply mimicking real people (Nina said her voices “are just imitating that person”).

##### Sub-Theme II: Making Sense of Voices

Probably influenced by societal representations and dominant mental health discourses, all but one of the women understood their voices as signs of a serious mental health problem. Sue said of the voices, “I could tell I was becoming ill.” Nonetheless, the participants used a mix of interacting external (negative life events) and internal (personal, inner life-based) explanations. For example, despite her conceptualization of voices as a sign of illness, Sue explained these as the result of an external event (a quarrel) and how she felt (guilty). Participants often searched through many possible explanations; Kim had moved from a psychological model (linking the voices to experiences of being abused) to a paranormal one, “but I know…that those voices are real. I know they are coming from a different paradigm.…” Yet, none of these explanations seemed completely satisfactory. The women were left perplexed and troubled, with Jo stating that “all the things that have happened to me in the past have been jumbled up with maybe things I’ve read, things I’ve watched, conversations I’ve had with people… have all been put in a big pot. They were the ingredients, and somebody’s mixed it with a spoon… and it’s just come out in that way.”

##### Sub-Theme III: Consequences of Hearing Voices

The women all portrayed hearing voices as a “knock-back” in all aspects of life. This included problems with work (Nina: “It’s been difficult to work… since I started hearing voices”), social problems (Helen: “I find it very difficult to get out and do anything really. Cause I’m so scared that people are gonna… ‘ugh’, you know, ‘that’s that strange mad woman’”), and money (Pat: “There’s no security”).

The voices also took an emotional toll, with Nina stating that it felt like she was being violated “like some kind of spiritual rape… the more I shouted at them the… the louder they got… that was just draining,” with reduced hopes for the future (Pat: “…if you dwell on it you think ‘well, I’m worth nothing now’, I can’t work again”) and loss of relationship and consequent isolation (Helen: “I lost a lot of friends”).

##### Sub-Theme IV: Coping

Apart from coping through a search for meaning, participants also had various ways of coping with their experience of voices. First, psychologically: for example re-storying negatives into positives (e.g., Jo: “like there were bad consequences but I’ve worked with it to make it into positives…”); lowering expectations to match perceived reduced abilities as a result of voice-hearing; constructing aspects of their experience as reasonable/logical; and, contextualizing feelings or experiences within particular circumstances and the broader social context. Second, practically pursuing activities that increased their sense of empowerment (Ann “…I’d like to go back to education […] it’s good to have a regular … a regular something”). And finally, through relationships, groups, and voluntary organizations that allowed a broader role, such as educating the public (Jo: “…the only thing I can do about [the stigmatisation of voice-hearing] it’s to try and break down the barriers. That’s one of the reasons why I got involved with [local voluntary organisation], because I feel that’s one way of coping with it.”).

#### Theme 2: Life Story

##### Sub-Theme I: Negative Family Relationships

Seven women reported experiencing negative family relationships in their upbringing. Sue felt her family were “entirely negative” and Kim stated that “my family are just shit, basically, horrible people.” They also reported experiencing constant tension, conflict and rejection. Nina stated that her mother had constant and seemingly unpredictable changes in mood and behavior, which was mirrored in her experience of voice-hearing – over which she felt she had no control either.

##### Sub-Theme II: Abuse

Seven women spoke of some kind of abuse in early life, including physical, verbal, and emotional abuse in the family home, bullying at school, and being subjected to physical or sexual assaults by men when adults. While for Kim her “evil” abusive voice was that of her abuser, for Helen and Nina the connection was indirect, in that abusive relationships or violent attacks had negatively affected their self-esteem in turn leaving them open to further abuse by voices (Helen: “…I was out one night and I was attacked and that, you know, I was just sort of getting more kind of confident and then that happened and it was right back to the beginning.”).

##### Sub-Theme III: Losses

Many of the voice-hearers had lost either family, close friends, or partners, and experienced difficult parental separation, often leading to feelings of loneliness or abandonment, and possibly reduced trust in others (Ann: “everyone I’ve ever met, or been with, they’ve always disappointed me. You know, like my dad, my step-mum, my uncles, my aunties”).

##### Sub-Theme IV: Never Being Good Enough

The life events above left the women with a feeling of worthlessness, often exacerbated by hearing critical or demeaning voices. In addition, the experience of constant and intense criticism from at least one parental figure from an early age (sometimes subtle and sometimes more direct) was commonly affirmed and mirrored how these women reported their experience of voices. Sue stated that “I think my relationship with my mother has always been one of me never measuring up to what she wanted, so that there was an intense criticism of me at all times.”

#### Theme 3: Gender and Wider Contexts

##### Sub-Theme I: Being Women Who Hear Voices

Seven participants related voice-hearing to being women, primarily in terms of the voice manifestation and origin, and the restriction or devaluation of their social positions. Three women portrayed voices and related phenomena as gender-specific in content and interaction, which seemed to reflect the women’s (subordinate) positions in social power relations. For example, Jo talked of the voices’ use of male-centered pornographic imagery and language, which as a woman she experienced as hostile and subjugating: “I…certainly don’t think some of the imagery and some of the voices would have say quite the sorts of things they did then … because they were quite detrimental sexually-wise […] so, [men] wouldn’t have that power struggle.”

Voice-hearing was also linked by two women to intuition, which was viewed as an intrinsically feminine quality. For Pat and Kim, because this extra-perception allowed access to another world or dimension, this was a benefit; they felt special. Kim described how “I think there’s something unique about being a woman, which is about intuition… that… men can’t have that. And I used to see it as a curse but now I see it as a gift. And I don’t think it would’ve been possible, to have that gift if I was a male.” However, most of the women spoke clearly about feeling that their gender had placed them at a disadvantage in the social context and hearing mostly negative voices or, at least negatively experienced voices, was an additional burden or disadvantage with which to contend. Helen stated that “I think it’s a toughie whether you’re male or female with voices, but if you’re up against it as a woman in the first place, then hearing voices ain’t gonna do you no favours (laugh)!”

##### Sub-Theme II: Being a Woman in Contemporary Britain

In addition to inequalities resulting from (stigmatizing) social and (pathologising) psychiatric discourses about voices, the women reported a contextual effect related to their gender, particularly in relation to social disempowerment and restriction – feelings paralleled in their accounts of their voice experience. Seven participants felt that women were generally seen as inferior to men in our society, as an “underclass.” From their accounts, the disempowerment of women in society seemed to be mainly achieved in three ways: through exclusion, stigmatizing language, and devaluation. Ostracism from social and cultural systems, like the Church, created a belief in one’s vulnerability. Pat stated that: “If I’d been a man I’d been brought up invested in, spiritually, instead of un-invested in. I would’ve been included in the communion of the church, instead of excluded and left to bring myself up.” This also occurred through language that represented them, for instance, as unintelligent and led by emotion, emotionality understood in society largely as a deficiency. Abusive language or undermining comments were similarly employed by voices (Kim: “there’s this other stuff about ‘stupid little woman’…[Male colleagues would] always be patronising, ‘you’re just overreacting’, that’s a classic”).

Discourses consistently discounted women and sent the message that they needed a man in their lives, regardless of personal or material achievements (e.g., a successful career). The implicit meaning of this suggestion, that they were not whole or worthy in their own right, echoing the voices’ messages (Nina: “…they encourage women “you’ve got to have a man, a husband to look after you”).

Finally, through sexualizing and objectifying women in a variety of settings, including the work place, as Kim described “I’d be in meetings with men, talking about really important things, and they’re just staring at my breasts, all of that stuff….” Unfortunately, linked to this last point was the experience of three women being subjected by men to physical or sexual attacks, in childhood and/or adulthood.

The women interviewed saw the abuse they had been subjected to in life, and by their voices, as closely related to their gender (Kim: “Well, I wouldn’t have been through all the sexual abuse [if she had been a man]. I know little boys get abused as well, but grown men don’t tend to get raped”). Only Kim talked about employing a strategy to get heard in a male dominated world, utilizing intellectual, maybe privileged “male” strategies, such as logic and scientific methods of discovery: “I mean, I’ve dealt with [being patronised] by just doing the job and being intelligent about it. I’ve got a [name] degree, I know how to put arguments together, and when people would come back with crap I would just keep saying where’s the evidence, where’s the evidence.”

Women reflected on feeling constricted, controlled, and pressured by their given roles and punished when they attempted to step out of bounds. For them, the twenty-first century woman may be allowed a broader range of roles in society, but a disproportionate pressure was exerted on them to succeed in all aspects of life: social (professional), personal (wife, mother, lover, carer, and housewife), and even physical (appearance and weight). However, in these women’s narratives men did not always enforce societal rules; other women served as agents of social control of women who were perceived as stepping out of feminine roles. Kim stated that “I’ve been treated quite badly by other professional women as well, and…and sometimes they’re really worse.”

Socio-cultural control was also reported in other aspects, such as emotional expression. It seemed the women felt their expression of strong emotion was not socially accepted, and so they had to find alternative, socially sanctioned means of expression. In some cases societal norms were more implicit, in regards to distress (Pat: “I was getting very upset because he wouldn’t forgive me certain things. I stormed out and stuffed myself – this is what I do when I’m upset”). Anger also appeared; Helen linked her little girl voice to her anger, “So yeah, that’s where she comes from. And the kicking and screaming, I really wanted to ‘grrr!’, and couldn’t, you know (laugh)…I used to very much turn it inwards, I used to self-harm quite regularly. Ammm…these days I tend to shove a cushion around or shout, scream and holler on me own, and that sort of gets it out of my system and then I’m alright after that, you know.” Regarding this last point, Kim highlighted how emotion too is gender-specific and socially defined, by calling into question the different labels attached to the same behavior in both genders: “I’d say something and be called aggressive, the male colleague next to me would be aggressive and people would not say he was being aggressive.”

### Discussion

#### Voices and Relationships

All of the women heard (or had heard) voices which were critical, controlling, angry, and used abusive language. Although some also described hearing “positive,” encouraging voices, they also experienced these as distressing because they conceptualized voice-hearing as a sign of illness, at least initially or at some level. While, more often than not, participants did not think there was a direct life analog to their voices, they did sound like or remind most of the women of someone personally known to them. Consistent with previous research (64, 65), the voice-hearer’s relationship with their voices reflected their relationships with other people in their social world. Voices were experienced much like important long-standing figures from early life, family members (particular mothers), and partners, who the women felt were implacably critical, blaming, controlling, and pressing for achievements. Undermining, abusive talk by voices sometimes echoed the very same words women had previously heard from others. This was similarly described in relation to attitudes; they had experienced both voices and others as mostly overpowering in the relationship.

#### Voices and Gender

Given voices are shaped by culture (66), voices’ talk, tone and practices were oppressive in ways that particularly related to the hearers’ gender. This was evidenced by women’s accounts revealing patriarchal attitudes in some voices and the devices voices used to overpower and control them. Participants’ questioning of their worth was also linked to wider misogynistic discourses. For example, the women described feeling dominated and subordinated by socially prescribed feminine roles. While they did not always try to conform to the stereotype of an all-encompassing, perfect woman, they distinctively talked about feeling the pressures in modern Britain to play these roles.

Resonating with Williams (67), the women linked feeling hurt and disempowered to societal practices such as stigmatization and discrimination. Social marginalization and inequalities seemed to affect their social status powerfully and negatively, as they talked about feeling socially devalued and forced into dependency on social systems of care like Mental Health and Social Services. As in previous literature [e.g., Ref. (68)], participants depicted mostly negative, serious, and enduring consequences to different aspects of their lives due to the experience of voices within a pathologizing culture. They considered social effects on their personal life to be largely detrimental in the material, psychological, emotional, and relational realms. Society’s historical silencing of women’s voices (69) was compounded by the social stigma and isolation attached to voices (70, 71). Therefore, these women had the socially added burden of being positioned between two difficult choices: “coming out” versus living “in the closet” (72). That is, the women were doubly silenced by discourses and attitudes, both about women and about voices, reflecting a double disadvantage.

#### Women’s Agency

In line with previous literature (73), the women’s resistance was evident in many ways, for example, while some women appeared at first glance to respond to the experience with helplessness and hopelessness (74), they storied a struggle to redress power-balance, fighting against voices with thoughts, spoken words, and even shouting back. They employed coping strategies that ranged from avoidance to taking responsibility and control over both voices (e.g., setting limits) and life, such as continuing to pursue their own desires regarding relationships, career, and leisure.

Despite an internalization of the illness discourse of voice-hearing – unsurprising given predominant pathologising medical discourses in our society (51) – the women held concurrent individualized meanings of the experience (multi-factorial model, psychological, paranormal, and technological explanations). Furthermore, in their accounts it appeared that they had contextualized their narrative of their experience of voices within their “sense of self,” their backgrounds, proximal, and distal influences – often implicitly but sometimes very well-articulated. Although they mostly experienced voices as internal, they had gained an understanding of their voices through all of these contexts and were able to story their voice-hearing experience in positive terms, as an opportunity for growth and development, to help and educate others, and even as a gift.

## A Quantitative Study of Women Hearing Voices

In addition to seeking a greater understanding of the nature and meaning of voice-hearing to women, we were also interested to compare the voices heard by male and female voice-hearers, and to explore if there were differences in the male and female voices that people heard. A quantitative methodology was deemed best suited to this. The first aim of this study was to examine whether men and women, in a psychiatric context, differed in four aspects of voice-hearing; affect, acoustics/form, interpretation and antecedents. We hypothesized that, in a help-seeking sample, the predominant voice(s) would be associated with negative affect, irrespective of gender, and that voice-affect would not differ between male and female voice-hearers. Given that the acoustics/form of voices is broadly stable in those distressed by voice-hearing trans-diagnostically (1), we also did not hypothesize that this would differ by gender. We hypothesized that men and women might interpret voices differently, given the role of socio-cultural factors in the interpretation of voice-hearing (75). We also hypothesized that men and women may have different rates of certain antecedents to their voice-hearing, given that women are more likely than men to develop psychosis following traumatic events (76, 77), and that men are more likely than women to develop psychosis after using cannabis (78). The second aim of this study was to examine if the affect associated with hearing male voices differed to that associated with hearing female voices. We hypothesized that, due to cross-cultural gender stereotypes in which women are rated higher than men in traits relating to warmth (79), and in-line with the large number of instances of medieval voice-hearing which often involved hearing loving and kind heavenly women’s voices (e.g., Mary, female saints), that female voices that people heard would be perceived as more affectively positive, and less affectively negative, than male voices.

### Method

The basis for this study was an existing dataset, facets of which have already been reported on [e.g., Ref. (80)]. All analyses performed here have not previously been reported. As this was an exploratory study, no corrections to alpha were employed, with the recognition that the results would be labeled as exploratory and the ensuing hypotheses would have to be tested in further confirmatory studies (81).

#### Participants

The dataset consisted of 197 individuals (65 women) with a mean age of 32.56 years (SD = 10.55, range 15–63), all of whom had both received psychiatric diagnoses and had experienced voice-hearing. Age at assessment did not differ between men (mean = 33.04, SD = 11.04) and women (mean = 31.58, SD = 9.48), *t*(195) = 0.91, *p* = 0.36. Age at the onset of voices also did not differ between men (mean = 23.23, SD = 11.38) and women (mean = 22.92, SD = 9.80). Schizophrenia was the most common principal psychiatric diagnosis (81%), with others being affective psychosis (14%), other non-organic psychoses (3%), and borderline personality disorder (3%). The majority of participants were taking dopamine blockers (41). Diagnoses were made using the Structured Clinical Interview for DSM-III-R (82). Diagnoses related to the time of interview, but voice-hearing experiences reported could be from the past.

#### Procedure

All participants were administered the Mental Health Research Institute Unusual Perceptions Schedule [MUPS: (83)], a semi-structured interview with questions organized into seven main sections: physical characteristics, personal characteristics, relationship, and emotive aspects, form and content, cognitive processes, personal perceptions, and psychosocial issues. Although the mean age of onset of voice-hearing was 23.2 years (9.5 years prior to interview), the MUPS typically focused on voice-hearing experienced during the most recent problematic experience of this phenomena, with interviewers focusing only on voices of which the participant could remember details.

##### Affect

Positive affect associated with the persons’ predominant voice(s) (Cronbach’s α = 0.90) was calculated as the sum of positive tone (MUPS item 12; gentle, loving, kind, friendly, and quiet items), content (item 22; helpful, guiding, affirming, inspiring) and feeling (item 32; comforted, not alone anymore, reassured, excited, inspired, happy). Negative affect associated with the persons’ predominant voice(s) (Cronbach’s α = 0.92) was calculated as the sum of negative tone (item 12; harsh, angry, authoritative, bossy, malicious, menacing, sharp), content (item 22; persecutory, abusive, obscene, intrusive, derogatory, accusatory, threatening, critical), and feelings (item 32; terrified, irritated, sad, helpless, hopeless, angry, anxious, agitated, depressed, intruded upon, overwhelmed, frightened, out of control, confused).

##### Acoustics and Form

Mental Health Research Institute Unusual Perceptions Schedule data was analyzed relating to the number of voices heard (MUPS item 9), frequency (item 4; rarely, occasionally, often, constantly), location (item 8; inside head, outside head, both), volume (item 11; too faint to be heard properly, whisper, soft, normal, loud, yelling/screaming, other), reality (item 27a; completely imaginary, vague, dream-like, somewhat real, very real), gender (item 15; female, male, both, hard to identify), whether they spoke in the first, second, or third person (item 16), whether they gave commands (item 25; never, rarely, sometimes, often), involved running commentaries (item 21; never, rarely, sometimes, often), and/or conversed about the hearer (item 19a; never, rarely, sometimes, often).

##### Interpretation

MUPS data was analyzed in relation to questions asking participants whether their voice(s) reflected thoughts they may have had (MUPS item 23a; yes, maybe, unsure, no), whether they felt the voices were coming from themselves or were an aspect of themselves (item 40d; not at all, a little, somewhat, quite a lot, completely), and whether, before they started hearing voices, they knew someone else who had had a similar experience (item 40i-i; yes, no).

##### Antecedents

Mental Health Research Institute Unusual Perceptions Schedule data was analyzed relating to whether participants reported experiencing any of the following events when the voices and/or sounds first started; stress, friendship problems, family problems, isolation (loneliness or social isolation), tiredness (sleep deprivation, exhaustion, or prolonged tiredness), physical illness, divorce, death of significant other, major trauma (e.g., rape, assault), life change (change in job, moved countries, financial situation change), change in medication/recreational drugs.

### Results

#### Differences between Male and Female Voice-Hearers

##### Affect

There were no differences between men and women in the positive, *t*(194) = 1.40, *p* = 0.16, or negative, *t*(194) = 0.80, *p* = 0.43, affect of their predominant voice(s) (Table 1).

**Table 1:** **Properties of voices**.

Variable	Men (n = 132)	Women (n = 65)
**Affect^a^**		
Positive voice affect	5.78 (4.72)	4.78 (4.57)
Negative voice affect	15.70 (7.80)	16.65 (7.77)
**Properties of voice(s)**		
Form of address^b^		
First person	24%	36%
Second person	68%	81%
Third person	62%	72%
Does not address	30%	25%
Voices conversing^a^		
Never	49%	31%
Rarely	5%	11%
Sometimes	26%	23%
Often	23%	35%
**Interpretation of voices**		
Voices reflect own thoughts		
No	39%	22%
Unsure	8%	5%
Maybe	9%	12%
Yes	44%	62%
**Antecedent events^c^**		
Stress	78%	83%
Friendship/relationship problem	55%	78%
Family problems	54%	64%
Physical illness	15%	38%
Medication/recreational drugs	39%	22%
Major trauma	32%	47%
Divorce	11%	6%
Change job/finance/location	57%	57%
Loneliness	69%	78%
Tiredness	59%	77%
Death of significant other	30%	36%

*^a^Missing data for one male participant*.

*^b^Missing data for three male participants and one female participant*.

*^c^Missing data for men ranged from 12 to 31 participants, depending on the question, and from 5 to 12 female participants*.

##### Properties of Voices

Women more often than men had voices that conversed with each other about them, χ^2^(3) = 9.69, *p* = 0.02 (Table 1). There were no significant differences between men and women in the frequency of voices which spoke in the first, χ^2^(1) = 3.01, *p* = 0.08, second, χ^2^(1) = 3.65, *p* = 0.06, or third person, χ^2^(1) = 1.84, *p* = 0.18 (Table 1). There were also no significant differences in voice frequency, χ^2^(3) = 1.73, *p* = 0.63, location, χ^2^(2) = 4.34, *p* = 0.11, reality, χ^2^(4) = 1.10, *p* = 0.90, gender, χ^2^(4) = 3.41, *p* = 0.49, or prevalence of running commentaries, χ^2^(3) = 4.26, *p* = 0.24, or commands, χ^2^(3) = 1.44, *p* = 0.70. For participants who were able to state how many distinct voices they heard, there were no differences between the number of voices heard by men (*n* = 95, mean = 4.32, SD = 5.46) and women (*n* = 47, mean = 4.19, SD = 3.68), *t*(140) = 0.14, *p* = 0.88.

##### Interpretation of Voices

Women were more likely to state that the voices reflected thoughts they may have had, χ^2^(3) = 7.86, *p* = 0.05, and to have known someone else who had experienced hallucinations before their own voices started, χ^2^(3) = 6.20, *p* = 0.01. There were no gender differences in whether the person agreed that the voice was coming from themselves or was an aspect of themselves, χ^2^(4) = 6.96, *p* = 0.14.

##### Antecedent Events

Women were more likely than men to report having experienced friendship/relationship problems, χ^2^(1) = 8.24, *p* = 0.004, tiredness, χ^2^(1) = 5.20 *p* = 0.02, and physical illness, χ^2^(1) = 10.47, *p* = 0.001, at the time of onset of voices. There was also a non-significant trend for women to be more likely to report a major trauma at the onset of voices, χ^2^(1) = 3.56, *p* = 0.06. In contrast, men were more likely to report medication/recreational drug use at the time of onset of voices, χ^2^(1) = 4.53, *p* = 0.03. There were no differences between the genders in rates of experiencing a change job/finance/location, χ^2^(1) = 0.01, *p* = 0.92, loneliness, χ^2^(1) = 1.68, *p* = 0.20, stress, χ^2^(1) = 0.63 *p* = 0.43, family problems, χ^2^(1) = 1.57, *p* = 0.21, divorce, χ^2^(1) = 1.12, *p* = 0.29, or death of a significant other, χ^2^(1) = 0.82, *p* = 0.37.

#### Differences between Male and Female Voices

Of the 169 participants who could identify the gender(s) of their voice(s), 5.3% heard only female voices, 26.6% heard only male voices, and the remaining 68.1% of participants heard both male and female voices (Table 2). There was no evidence that men and women differed in the gender of voices they heard, χ(4) = 3.41, *p* = 0.49. Comparing participants who heard only female voices (*n* = 9) with participants who only heard male voices (*n* = 45) on the continuous variables of positive and negative voice-affect would only have had satisfactory power (β = 0.80) to detect very large effect sizes (Cohen’s *d* = 1.04) at α = 0.05 (two-tailed). Such comparisons were deemed too underpowered to be informative. However, power calculations indicated that comparing participants who only heard male voices (*n* = 45; Voices_M_) with those who heard both male and female voices (*n* = 115; Voices_M+F_) had satisfactory power (β = 0.80) to detect medium effect sizes (*d* = 0.50) at α = 0.05 (two-tailed). We hence undertook this latter type of analysis which, although not offering a direct test of our hypothesis that male and female voices would differ affectively, was deemed still likely to offer suggestive findings, and offer fruitful hypotheses for further research.

**Table 2:** **Gender of voices heard by participants, split by gender of participant**.

	Gender of voice(s) heard				
Gender of hearer	Male	Female	Male and Female	Unclear	Total
Female	11	3	39	12	65
Male	34	6	76	16	132
Total	45	9	115	28	197

There were no differences between Voices_M_ and Voices_M+F_ in negative affect associated with the predominant voice(s), *t*(157) = 1.00, *p* = 0.32. However, Voices_M+F_ had significantly greater levels of positive affect associated with their predominant voice(s), *t*(157) = 2.02, *p* = 0.04, than Voices_M_. In order to examine whether specific types of positive content, tone or feeling were driving this effect, we examined these individually. Voices_M+F_ were more likely to have a predominant voice(s) with a loving, χ^2^(1) = 6.80, *p* = 0.009, and kind tone, χ^2^(1) = 8.79, *p* = 0.003, which made them feel happy, χ^2^(1) = 3.85, *p* = 0.05, or inspired, χ^2^(1) = 4.10, *p* = 0.04, and was affirming, χ^2^(1) = 4.72, *p* = 0.03.

### Summary

The affect and form of voices heard by men and women were very similar. The exception to this was a greater rate of hearing voices conversing in women. Gender differences were more pronounced for the interpretation and antecedents of voice-hearing. Women were more open to the possibility that their voices reflected their own thoughts, and more likely to have known someone else who had experienced hallucinations before their own voice-hearing started. Women were also more likely to report friendship/relationship problems, tiredness, and physical illness at the time of onset of voices, whereas men were more likely to have been using medication/recreational drug use at voice-onset. People who heard male and female voices had a predominant voice with greater positive affect than those who only heard male voices. Given that we did not correct our significance level for multiple testing in this preliminary study, these results should be seen as exploratory, with the need for replication before firmer conclusions can be drawn.

## Integration of Findings and Future Directions

### Connections between Historical and Contemporary Women’s Voice-Hearing

Our historical review, as well as our contemporary qualitative and quantitative investigations, found that the form, content, interpretation, and antecedents of voice-hearing appeared to be influenced by gender, presenting women who hear voices with specific challenges. The findings of our studies of contemporary women’s experiences of voice-hearing echoed, in a variety of ways, women’s historical experiences of voice-hearing.

The sentiment that women were incomplete beings, seen in the medieval and early modern period, was reproduced in the messages that contemporary women received from both society and their voices; both implied that they were not whole or worthy in their own right. Similarly, contemporary women voice-hearers reflected on feeling constricted, controlled, and pressured by societally prescribed gender-roles, and being punished when they attempted to step out of bounds. As this treatment of women was mirrored in the voices they heard, some women’s voices could be seen as internalizations of continuing sexist societal attitudes toward women. This would be consistent with a body of research that has found that people’s existing thoughts and ideas may come to be reflected in the content of the voices they hear (84), that derogatory voice-content is linked with depression (85), that voice-hearer’s relations with their voices reflect their relationships in their social world (64), and that the content of voices can typically be related to the socio-emotional past and present of an individual (86).

In the medieval period, women’s experiences of voice-hearing were defined and interpreted by men. The overwhelming majority of contemporary women in our qualitative study had their voices defined and interpreted by medicine. Yet none of the understandings the women had been offered adequately explained to them their hearing-voices; all of the women had been left perplexed and troubled and continued searching for explanations. Encouraging women voice-hearers to develop their own ways of understanding voices, and validating these, such as through the HVM approach, could allow women to find meaning in their experiences, regain agency, and grow from their experiences (49). Yet, medieval women visionaries got something important from their male clerical scribes, i.e., their approval and support of their visions, and we may ask if modern voice-hearers get something similar from researchers and medical professionals. For example, in what ways do medical professionals still practice “discernment of voices”? Whereas medical professionals may discern voices as a symptom of illness, which some may find comforting despite the potential othering and stigmatization implicit therein; voice-hearing groups may offer a more normalizing perspective that some women may find more useful in supporting their agency in their mental health and lives. Multiple routes to recovery are possible (87).

Historically, voice-hearing has sometimes served as a way for women to make societal criticisms that their own voices alone would not be given the authority to convey. In a parallel to this, our qualitative study found that some women heard voices that they believed reflected emotions that society deemed inappropriate for women to express, in particular, anger outwardly expressed. Campbell (88) has argued that, even though aggression by women occurs more rarely than aggression by men for a range of evolutionary reasons, patriarchal institutions have stigmatized aggression in women. Men’s aggression is treated as a natural expression of male competitiveness, often being valorized, yet women’s aggression is “rendered unnatural and treated as evidence of pseudo-masculinity or irrationality” [Ref. (88), p. 214] stemming from mental illness/hormonal imbalance. Indeed, a woman appearing before a criminal court is approximately twice as likely as a man to be dealt with by psychiatric rather than penal means (89). Why patriarchy leads to this is unclear, but proposals include serving to maintain female dependence upon men for protection, excluding women from warfare and from consequent political power, and deflecting attention from the fact that much female aggression is responsive to men’s domestic abuse (88).

As a result, men are more likely than women to describe their involvement in aggression in justificatory terms, viewing it as a form of legitimate social control over others’ misbehavior e.g., “aggression is necessary to get through to some people” (90–92). In contrast, women are more likely to describe their involvement in aggression in exculpatory terms employing an expressive representation which denies their full responsibility for their actions, e.g., a regretted loss of self-control caused by high levels of stress (90–92). For women, anger and aggression come to represent a loss of self-control, for which they are made to feel ashamed. Given this, one interpretation of our finding is that the voices heard by some women are internalized expressions of what society deems gender-inappropriate affect, that is, the voices express what women cannot express in their own right. One way to experimentally address these hypotheses would be through facilitating spaces/conversations for women to safely express emotions ostensibly underpinning their voices, and examine if this led to change in their voices (in terms of frequency or content).

Trauma survivors have constantly employed creative strategies in order to survive, and behavior labeled by others as bizarre, unacceptable, or even threatening, has served a protective purpose. The function of dissociation has often been seen as a way to continue to obtain care and physical sustenance from caregivers in childhood or in other situations of dependency (93, 94). Cross-cultural study has lead Bourguignon (95) to argue that “Acting out the identity of spirits in ritual possession trance offers women an acceptable, and consciously deniable, way to express unconscious, forbidden thoughts and feelings, particularly in situations of social subordination” (p. 558). Just as medieval instances of voice-hearing were argued to sometimes be the expression of socially unacceptable content, voice-hearing today may in some cases be the expression of experiences that society does not wish to acknowledge. Such a hypothesis requires further research.

### Directions for Further Study of Differences in Hearing Voices Among Genders

Although the predominant voice(s) heard by contemporary men and women were similar in terms of their overall levels of positive and negative affect, people who heard both male and female voices had a predominant voice with greater levels of positive affect (e.g., rated as more loving and kind), compared to people who heard only male voices. We were unable to directly test our hypothesis that female voices are associated with more positive affect than male voices due to a lack of statistical power. Potential confounds in concluding from our findings that hearing female voices is associated with more positive affect than hearing male voices include that simply hearing more voices *per se* may be associated with the creation of a predominant voice(s) with a greater level of positive affect. Speculatively, this could arise as a dialogic response to an initial, affectively negative voice. Nevertheless our finding offers a hypothesis for future study, which could attempt to establish if social representations and gender-stereotypes of women are reflected in the intra-psychic realm of voice-hearing. Cross-cultural studies would be of particular value here.

It was notable that our historical review of medieval women’s voice-hearing experiences was primarily limited to a specific type of account that had deemed worthy of recording and preservation at the time (linked to religion). Our contemporary qualitative study was also limited by the specific type of accounts that we elicited and recorded, which were from women who had been in contact with mental health services. More generally, didactic medical case-studies are skewed toward individuals who are more easily understood by clinicians re-telling the story, and individuals speaking the same language and born in the same country. There is hence the need to examine accounts of voice-hearing from women who have not come into contact with mental health services, and from a range of backgrounds, to examine how they have managed to navigate their voice-hearing.

Just as medieval male scribes shaped the narratives of women voice-hearers, our previous assumptions and knowledge shaped the analysis of participants’ accounts in the qualitative study, and the choice of questions employed by the MUPS shaped the quantitative picture of voice-hearing that emerged. This highlights the need for participant-led research in which the important aspects of the experience to voice-hearing women form the focus of inquiry. This also raises a more general point about how mental health professionals can promote women’s own definitions of their voice-hearing and ownership over their mental health and lives, given the range of discourses available to account for voice-hearing. As Hornstein (55) has argued, respecting the diversity of experience will mean making room for perspectives that are disconcerting to the traditions of mental health professionals. This may also include allowances for participants to identify as other than cisgendered. Individuals identifying somewhere on the gender fluidity spectrum are commonly under-represented in research. Individuals who identify with under-represented sexual orientations also experience unique challenges and experiences of trauma that deserve recognition and better understanding, and almost all individuals with non-dominant gender or sexual expression struggle with access to appropriately sensitive services (96). Studies examining the experiences of under-represented ethic and cultural groups’ experiences of voice-hearing and the interplay of racism, xenophobia, and stigma along with gender expectations and misogyny would also be extremely valuable to understanding the medical system’s responsibility to respond to colonizing impulses or structures.

### Precursors and Adverse Life Experiences

Our qualitative study highlighted links between early life abuse and voice-hearing. Our quantitative study not only echoed this, but found a trend toward women being more likely to report a major trauma at the onset of voices than men, and also found that women were more likely than men to have had emotional (friendship/relationship) and somatic (physical illness, tiredness) problems at the onset of voices. In contrast, men were more likely to report medication/recreational drug use at the time of onset of voices. The former finding was consistent with previous work showing women being more likely than men to develop psychosis following some traumas (76, 77). The latter finding was consistent with previous work which has found men being more likely than women to experience psychosis following cannabis use (78). However, our finding that socio-emotional and somatic problems were more common antecedents of voice-hearing in women was a novel finding. Women may have a different trajectory into voice-hearing to men. Alternatively, both men and women may experience trauma at the onset of voices, but men fail to report it, instead only reporting a sequela of the trauma; medication/drug use. This highlights the need for more in-depth trauma screening.

In terms of the link between abuse and voice-hearing, Hornstein (55) has argued that feminist psychologists have not had the same enthusiasm for a woman’s right to her mental life as for her right to her physicality. Yet, one’s mental life is determined, in part, by one’s physical life. Given the relation between trauma (e.g., rape) and voice-hearing, if women’s rights to their physicality were un-impinged then the prevalence of distressing mental experiences, including voice-hearing, should reduce significantly. Indeed, it has been estimated that if five specific types of child abuse were ended, this would remove around a third of cases of psychosis (97).

Such findings raise questions about the nature of schizophrenia itself. As has been argued elsewhere (98, 99), it is likely that a subset of people diagnosed with schizophrenia who hear voices may be experiencing a specific form of post-traumatic reaction, differentiable from other characteristic forms of post-traumatic reaction that may accompany voice-hearing such as experiences and behaviors detailed in the diagnostic criteria for borderline personality disorder or PTSD (100).

### Compounded Oppression

Women who hear voices and have a history of childhood and/or adult sexual victimization [a common combination; (101)] are likely to suffer a threefold prejudice against testimony given by them. First, sexism has led to the testimony of women historically being viewed with suspicion in the legal realm (102). Second, being deemed mentally ill as a result of experiences such as voice-hearing can also be used to challenge the credibility of testimony given in court (103). Historically, women and the insane have been given as exemplars of people unable to give reliable testimony, along with children, slaves and criminals. Third, the testimony of women in relation to reporting sexual abuse has been, and continues to be, viewed as suspect in the legal realm. This is due to sexism and untenable stereotypes resulting from factors such as laws traditionally formed by men that hold unchallenged, biased views about women, and a lack of women amongst decision makers within the judicial system (102). Thus, a female voice-hearer with a history of sexual abuse faces a unique challenge to have her experiences heard and believed.

Given the relationship between women voice-hearers and trauma, abuse, oppression, and marginalization, the work in this paper connects to a wider body of literature within cultural and feminist theory which politicizes the category of trauma in an attempt to form a trauma culture “that doesn’t involve medical diagnoses or victims” [Ref. (104), p. 1]. This attempts to avoid trauma being experienced as individualized shame, psychopathology and misery, and to instead situate it within a larger context (105). As Cvetkovich argues, “trauma forges overt connections between politics and emotion” [Ref. (104), p. 3]. Cvetkovich’s focus is lesbian, gay, bisexual, and transgender forms of activism which have attempted to publicly contest shared traumatic histories (such as OUTrage and ACT UP) and to provide an archive of objects, such as flyers, posters, banners, testimonials and memoirs, which attest to these shared traumatic and often silenced histories. Importantly, Cvetkovich argues these are not just archives of objects, but also archives of *feeling*; they transmit and enact some of the trauma that connects the past with the present, staging affective experiences that, as she argues, “can provide the basis for new cultures” (ibid, p. 7). These performative strategies link a growing body of work across cultural theory, which may hold important insights for future work exploring the phenomenology of voice-hearing, going beyond the interview form of research presented here.

Women, more often than men, had voices that conversed with each other about them. If not a Type I error, this could reflect the long-term objectification and devaluation of women in our culture, with women being objects that are talked about, rather than subjects with whom to talk. This is consistent with our qualitative finding that women voice-hearers experienced abusive, infantilising discourses, which treated them as objects. Furthermore, female–female violence more often involves indirect aggression [e.g., ostracism, verbal harassment, rumor spreading; (106)] as opposed to direct attacks (e.g., physical violence) more typical of male–male (and male–female) violence (107), hence, women’s experiences of attacks from voices may be more likely to take this form. One way to cope with such objectifying, conversing voices may be to make oneself a subject, not an object. A previously accepted diagnostic heuristic was that voices that could converse with the therapist and the patient were deemed dissociative, while those which could not be engaged relationally were deemed psychotic (108). More recent research not only questions this distinction [e.g., Ref. (98)] but also suggests that engaging voices relationally is not a characteristic, but a skill that may be learned, honed, and perhaps taught by clinicians or peers to beneficial effect (109). If as has been proposed, voices have their roots in a process related to dissociation (109) dialog with voices may help re-integrate such experiences, although traumatic events are notoriously difficult to integrate into the totality of one’s life experiences (33). This area remains poorly understood.

In the medieval period, the value of women’s voice-hearing experiences was in part judged by their adherence to socially prescribed gender norms. In a medical model, the content of the voices is often deemed irrelevant, leaving less space for women voice-hearer’s deviations from gendered norms to impact upon the assessment of their voices specifically. However, it was clear that the women felt judged and controlled by gender expectations even if the content of their voices was not scrutinized. Whether women who hear voices and breach gendered norms are more likely to be given a psychiatric diagnosis, or more likely to be given a specific type of diagnosis than women who hear voices and comply with gender norms, remains to be investigated.

In conclusion, it appears that sexism, sexist exploitation, and oppression (10) have engendered voice-hearing in women, and influenced aspects of its form and content. Despite, at times, deliberate obfuscation of abuse as a causative agent for voice-hearing, this continues to re-emerge in the narrative of psychiatry as women continue to assert the validity of their experiences. Patriarchy is an overlooked structural factor in relation to voice-hearing, and a greater focus on this, as well as other distal causes (110), is long overdue. As we have demonstrated here, such a project is likely to benefit from interdisciplinary study and multiple research methodologies.

## Author Contributions

All authors significantly contributed to either the conception, performance, analysis, or interpretation of the study. All authors contributed to the final version of the manuscript.

## Conflict of Interest Statement

The authors declare that the research was conducted in the absence of any commercial or financial relationships that could be construed as a potential conflict of interest.
